# Benchmarking Environmental Health Influences on Food Security in Very Remote Indigenous Communities in Australia: Protocol for a Mixed Methods Study

**DOI:** 10.2196/71697

**Published:** 2025-12-24

**Authors:** Melissa Stoneham, Miranda Hendry, Scott Mackenzie, Christina Mary Pollard

**Affiliations:** 1 School of Population Health Curtin University Perth Australia

**Keywords:** food security, remoteness, housing, environmental health, diet, Indigenous peoples, Aboriginal and Torres Strait Islander, nutrition, price, sanitation

## Abstract

**Background:**

Many factors including the impact of colonization and subsequent intergenerational trauma contribute to health inequalities for Aboriginal and Torres Strait Islander people, respectfully referred to as Indigenous Australians. The unacceptable health gap is higher for the Indigenous Australians living in very remote communities. Food insecurity—a lack of regular access to safe, nutritious, and affordable food—is influenced by both housing and retail environments. Ensuring that houses have functional and adequately maintained kitchens and access to affordable, healthy food are significant policy challenges for Australian governments; yet, little is known about these environmental health drivers in very remote areas.

**Objective:**

This study aims to benchmark environmental health food security risk factors impacting 19 very remote Indigenous communities in Western Australia. Specific objectives include using digital apps (1) to assess the appropriateness and suitability of kitchens in houses (internal environment), (2) to assess the affordability of food and sanitary goods (external environment) compared with the nearest town and capital city, and (3) to identify residents’ perceptions of appropriate kitchens.

**Methods:**

The mixed methods eHealth study includes 3 approaches. The internal environment is assessed via an in-house audit of facilities used to prepare, store, and cook food to maintain Healthy Living Principle 4 using a customized digital app and a 5-minute face-to-face yarn with tenants (n=130). This provides lived experience perspectives to inform housing and store pricing policy recommendations. The external environment assesses retail practices and food item (n=97) and sanitation product (n=28) prices in remote community stores, extending Healthy Diets ASAP (Australian Standardized Affordability and Price) to compare the mean price per product, the whole diet, and sanitation goods with the nearest town and capital city. Descriptive statistics and frequencies will be reported for the audits, and thematic analysis of the interviews will be undertaken.

**Results:**

Tenant interviews and data collection for the in-house and retail audits across the 19 communities will be undertaken by mid-2026, and the analysis will be completed by the end of 2026. Findings will be collated and triangulated to provide benchmark data for environmental health determinants of food security in very remote Western Australian communities. Preliminary findings will be shared with each community to support their advocacy, policy, and practices for timely maintenance of homes, suitable kitchen design, and store retail practices.

**Conclusions:**

This is the first study in Australia to explore the environmental health drivers of food insecurity in very remote Indigenous communities using digital technology from the perspectives of the tenant, in-house facilities, and in-store retail practices. The food security environmental health benchmarking will provide evidence for advocacy to promote culturally appropriate and practical solutions to improve living conditions and health of families in these areas.

**International Registered Report Identifier (IRRID):**

PRR1-10.2196/71697

## Introduction

### Context

This study is contextualized in very remote Australia and recognizes and respects Aboriginal and Torres Strait Islander people as custodians. This is acknowledged as a human right under the United Nations Declaration on the Rights of Indigenous Peoples [1]. Hereafter, the term “Indigenous” is used to refer respectfully to Australia’s First Peoples, also known as Aboriginal and Torres Strait Islander Peoples and First Nations Peoples [2].

### Background

The impact of colonization and the subsequent intergenerational trauma have resulted in an unacceptable health gap between the Indigenous and non-Indigenous Australian population [3], particularly for those people living in very remote communities. Health issues are also underpinned by geographical isolation, poor housing, socioeconomic disadvantage, and poor health literacy [4]. A specific health issue affecting many people residing in remote Indigenous Australian communities is food insecurity, driven by food stress resulting from low household income [5], coupled with a lack of access to affordable, nutritious, fresh food [6,7] and functional kitchens within homes to safely store, prepare, and cook food.

The research emerged from priorities identified by the Aboriginal or Torres Strait Islander sector. In 2022, the national Aboriginal health peak body (National Aboriginal Community Controlled Health Organisation) recommended that any policies or programs addressing food security be implemented in accordance with the 4 Priority Reform Areas of the National Agreement on Closing the Gap. This research aligns with these reform areas [8].

Western Australia was selected for this study, as it is the largest Australian state and has over 12,000 people living in over 200 remote Aboriginal communities [9]. With 89,000 people who identify as Aboriginal and Torres Strait Islander, 3.3% of Western Australia’s population, the state comprises higher than the national average (3.2%) [10]. Only remote communities were included in this research, as housing conditions for Aboriginal families living in urban areas are not equivalent or comparable due to access to services. In general, life expectancy is lower in remote areas, with Aboriginal people living in cities expected to live around 5 years longer than those living in remote and very remote areas [11].

### Cultural Determinants of Health

The cultural determinants of health and social and emotional well-being in Australian Indigenous peoples are protective factors that enhance resilience and strengthen, identify, and support health and well-being. These include a connection to country, family, kinship and community, language, cultural expression and continuity, self-determination, and leadership [12]. Almost a third of Indigenous Australians resided in remote or very remote areas in 2024 [13]. Over 89,000 people identify as Aboriginal or Torres Strait Islander in Western Australia, representing 3.3% of the population [10]. Geographically, Western Australia is the largest Australian state (the Aboriginal Lands Trust estate covers over 23 million hectares, 8.7% of the state), with 12,000 people residing in 200 remote Aboriginal communities [10,14,15]. There is a dearth of information available about the environmental influences of household food security in very remote communities in Western Australia.

### Food Security

Food security is a priority for public health and critical for good health. United Nations Sustainable Development Goal 2—ending hunger in all its forms to achieve food security and improve nutrition and sustainable agriculture, recognizes challenges for people living in geographically isolated communities [15].

Defined at the individual and household level, food security exists when “all people, at all times, have physical and economic access to sufficient, safe, and nutritious food to meet their dietary needs and food preferences for an active and healthy life” [16]. The converse food insecurity exists when “the availability of nutritionally adequate and safe foods, or the ability to acquire acceptable food in socially acceptable ways is limited or uncertain” [17].

In Australia, the prevalence of food insecurity is higher among Indigenous people residing in remote areas than those living in urban areas [18]. The National Aboriginal and Torres Strait Islander Health Survey conducted in 2022-2023 found that 41% of households had experienced food insecurity in the last 12 months, and just over half (51%) of the households were in remote areas. Almost a quarter (24%) of these households had experienced severe food insecurity compared to 17% in nonremote areas [12]. For many people residing in remote Aboriginal communities, the ability to buy, store, and cook healthy and nutritious food is limited, sometimes impossible. When combined with poor housing conditions, inadequate maintenance or failure to repair houses, and essential health hardware needed to store, cook, and prepare food such as stoves and refrigerators, the community’s efforts to achieve food security are undermined. Remote homes struggle to have access to affordable, continuous electricity and affordable, reliable access to safe-to-drink potable and palatable water [19].

Theoretical frameworks regarding the determinants of food insecurity have continued to evolve over time. The High-Level Panel of Experts on Food Security and Nutrition identifies 6 dimensions of food security including availability, access, utilization, stability, agency, and sustainability as essential. External resources are required to ensure food security, including clean water and sanitation, as “food must be available; households must have access to it; they must utilize it appropriately; and the whole system must be stable” [20]. The dimensions of agency and sustainability are particularly important in remote Indigenous communities and need to be aligned with Indigenous peoples’ right to self-determination and leadership. Agency refers to “the capacity of individuals and groups to exercise a degree of control over their own circumstances and to provide meaningful input into governance processes” [21]. Information regarding inequities in the food system is essential to support agencies at the individual and community level to address imbalances of power and to influence policy to address food insecurity. Sustainability refers to “food system practices that contribute to long-term regeneration of natural, social, and economic systems, ensuring the food needs of the present generations are met without compromising food needs of future generations” [22]. Many remote community stores have failed to operate as viable businesses, in part due to small market share and the cost and complexity of transport logistics [23]; yet, no regular real-time price monitoring occurs. Sustainable food systems need to be supported by strong government policy informed by timely, relevant research.

The causes and consequences of food insecurity are numerous, interconnected, and far-reaching. They include economic, social, and environmental circumstances influencing the ability to purchase, prepare, and use food; food availability and production; geographic isolation; transport logistics; climate events; the availability of food preparation, storage, and cooking facilities; and food literacy skills [24-26].

### Environmental Determinants of Food Insecurity—Food Access and Affordability

In 2024, over 200 food stores service remote Indigenous communities across Australia, providing 90% to 95% of the food consumed, and traditional foods contribute only a small, but important, supplement to people’s dietary intake [19]. Community stores are either managed by store management companies, where external agencies maintain control, or are community-owned and managed. Often, community-owned stores serve smaller populations and have less purchasing power or ability to buy in bulk. Residents in communities with no store are required to travel many hours on unsealed or seasonally inaccessible roads to a retail provider in a regional town to purchase their food.

Restricted access to and the limited choice for a variety of healthy foods in remote stores have negatively impacted the dietary habits and health of Indigenous people living in remote communities [27]. The National Indigenous Australians Agency (2025), following consultation across these areas, reported that people in these communities often relied on “cheaper convenience foods, which are often nutrient-poor and higher in calories,” as they were more affordable in these community stores [19]. Regular consumption of this food-insecure dietary pattern increases the risk of diet-related disease among Aboriginal and Torres Strait Islander Australians including diabetes, oral health issues, kidney disease, cardiovascular disease, and some cancers [19].

The prevalence of food insecurity in remote Indigenous communities and the role of community stores have been acknowledged for 30 years, with considerable policy inertia described in submissions to the numerous Parliamentary Inquiries over that time. In 2003, Food North, a government-commissioned report, identified the barriers and enablers to policy recommendations to address food insecurity in remote Indigenous communities across Australia [28]. Challenges identified related to transport logistics and supply change efficiencies, such as climate and weather events reducing the supply, quality, and affordability of food [28]. The lack of retail store viability in geographically isolated locations was described by remote community store managers [6,29]. Unregulated pricing of essential food items in these communities has been reported [28,30]. The 2009 House of Representatives Aboriginal and Torres Strait Islander Affairs’ Committee Inquiry into remote Aboriginal and Torres Strait Islander community stores recommendation number 29 was that store policy should require the display of pricing and cross-subsidies to promote healthier foods [28].

In 2020, a Parliamentary Inquiry into food price gauging identified that although food costs are very high in many remote communities, there was no evidence of systemic price gouging taking place in remote community stores [31]. In addition, the inquiry recommended measures to improve governance and oversight of community stores. In 2022, the Western Australia Committee on the Commissioner for Children and Young People initiated an inquiry into the most effective ways for Western Australia to address food insecurity for children and young people affected by poverty, resulting in 24 recommendations [32]. The government response was to recommend the Health and Education Department support school lunches and breakfasts, the creation of a Food Stress Index map to guide interventions, and for the Premier to take responsibility for food relief [33]. Despite some submissions highlighting environmental barriers to food security in remote communities, there was little regard for these or the cultural determinants of health. However, the Australian government’s National Strategy for Food Security in Remote Aboriginal and Torres Strait Islander Communities 2025-2035 highlights both these areas [19].

### Environmental Determinants of Food Insecurity—Housing and Health Hardware

Housing conditions for the Indigenous Australian population often differ greatly from those of the non-Indigenous Australian population. The households are larger in size, more likely to include a lone parent, and frequently comprise multigenerational members of extended family and kinship networks [20]. The scarcity of housing in remote Indigenous communities is a challenge. Crowded housing has been widely documented over several decades and is seen as a major contributor to, and symptom of, Indigenous disadvantage [34,35].

In remote Indigenous communities, food insecurity [36] and poor hygienic conditions of bedding and sleeping areas have been associated with increased disease transmission [37], and overuse of showering, washing, and kitchen facilities (health hardware) due to the higher number of tenants leads to breakages [38]. These are examples of health-related disadvantages in crowded remote houses. Policies implemented to construct and maintain remote housing have been found to be suboptimal for many years [39,40].

In addition to an insufficient housing stock in remote areas, the houses themselves deteriorate quickly [41] due to harsh climatic conditions, overcrowding, poor maintenance, and, in some cases, antisocial behavior [34,42,43]. Overcrowding also results in significant “wear and tear” on homes, driving up home maintenance costs. A lack of functional health hardware and the challenging internal living conditions of many houses resulting from unreliable access to maintenance are major concerns. An environmental health response to internal house living conditions aligns with the 9 Healthy Living Principles (HLPs) considered essential in a home to support personal health [44]. HLP 4 considers nutrition and reinforces the importance of functioning health hardware. Health hardware refers to the equipment or facilities in the house necessary to carry out healthy living practices, such as washing people, washing clothes, removal of human waste, and preparing food [44,45]. In 2018-2019, a small study found that 9% of First Nations households had no access to working facilities for food preparation, 4% had no access to working facilities to wash clothes and bedding, and 3% had no access to working facilities to wash household residents [46]. Nonfunctional health hardware has been associated with food insecurity and poor health in remote communities. Ali et al [47] found that poor remote housing maintenance and functionality were associated with gastrointestinal infections and skin, ear, eye, respiratory, and gastrointestinal illnesses. Quilty et al [48] found that homes lacking structural integrity, appropriate design, and insulation were more expensive to keep warm or cool or to operate essential health hardware (eg, refrigerators and stoves), leading to food scarcity and food insecurity. The last major survey of Indigenous housing quality in remote Western Australia was over 2 decades ago, a gap this research aims to fill [49].

The harsh climatic condition in many Australian remote Indigenous communities means that families spend time outside of their homes to escape overheated houses. Outdoor living spaces with suitable shading can reduce heat exposure and protect residents from the elements while meeting Indigenous people’s kinship [50] and lifestyle needs [34]. O’Rourke and Nash [51] explored the uses and functions of yards in Indigenous housing in semiarid Queensland and the Northern Territory and found that 73% of Indigenous families expressed a preference for outdoor shaded areas over internal rooms. Specifically, for food security, it has been demonstrated that design around a central open or covered cooking area that enables the preparation of traditional food and cooking methods is preferred in remote Indigenous communities [52,53]. Open cooking areas also reduce odors and food waste problems within the house, allow for the variable size of the eating group, and reduce smoke, grease, and vermin damage to cupboards in the kitchen area [53].

Recent assessments of the health consequences of poor housing and yard use in remote Indigenous communities have largely focused on infectious diseases and comfort [47,54,55]. However, since it has been over 20 years since the last major survey of Indigenous housing quality in remote Western Australia, this study is urgently called for. Low participation rate in food security research in remote areas of Western Australia is another challenge [36] and a gap, which this study intends to fill.

This study aims to use innovative digital methodology to explore and benchmark the environmental health determinants of food insecurity in remote Indigenous communities, providing important information to the community for policy and advocacy purposes. Specific objectives include using digital methods (1) to assess the appropriateness and suitability of kitchens in houses (internal environment), (2) to assess the affordability of food and sanitary goods and retail practices (external environment) compared with the nearest town and capital city, and (3) to identify residents’ concerns and perceptions of appropriate kitchens (internal environment).

## Methods

### Ethical Considerations

The Western Australian Aboriginal Health Ethics Committee granted ethics approval (HREC1370), and the Curtin University Health Research Ethics Committee granted reciprocal ethics approval (HRE2024-0727). As this research works alongside Indigenous practitioners and communities, the CONSIDER (Consolidated Criteria for Strengthening the Reporting of Health Research Involving Indigenous Peoples) was used to strengthen research practices and reporting to enhance research conduct and dissemination to support Indigenous health equity [56]. In addition, the research is consistent with the National Statement on Ethical Conduct in Human Research 2023, National Health and Medical Research Council’s *Ethical Conduct in Research With Aboriginal and Torres Strait Islander Peoples and Communities: Guidelines for Researchers and Stakeholders* [57]. The research partnership agreement includes protection of Indigenous intellectual property and knowledge arising from the research, including financial and intellectual benefits generated in line with the Our Culture: Our Future report. The project also follows Indigenous Data Sovereignty protocols that ensure that data on or about Indigenous peoples reflect their priorities, values, cultures, worldviews, and diversity. After outlining the research, participants give informed consent. There is no compensation for participation.

### Study Design

This mixed methods study aims to benchmark environmental health food security risk factors impacting 19 very remote Indigenous communities in Western Australia by auditing the appropriateness and suitability of kitchens in houses (internal environment), assessing food affordability and retail practices (external environment), and identifying residents’ concerns and perceptions of appropriate kitchens. A key objective of this unique, practice-informed study is to establish a baseline for environmental health factors influencing household food security in remote Indigenous communities in Western Australia. Measuring and reporting on the 3 aspects of household food security together—household kitchen functionality, community kitchen preferences, and external retail practices—have not been previously undertaken.

### Setting and Recruitment

This mixed methods study will be undertaken in 19 very remote Indigenous communities across 4 regions of Western Australia: the Pilbara, Goldfields, Midwest, and Central Desert regions. Very remote areas, as classified by the Australian Statistical Geography Standard-Remoteness Area classification, rely on the Accessibility/Remoteness Index of Australia, measuring the relative geographic access to services. Very remote areas [58] represent high levels of disadvantage based on the Australian Bureau of Statistics’ Index of Relative Socio-Economic Disadvantage for Areas [59]. People living in very remote areas are 58% more likely to speak an Australian Indigenous language at home [60]. The research will be undertaken alongside Curtin University’s #endingtrachoma project, which has established relationships with these communities. The #endingtrachoma project has been servicing Western Australia’s remote communities since 2019 and is delivered in partnership with Indigenous environmental health practitioners (EHPs) in each community. The project facilitates an approach led by the community including in-home bathroom and laundry assessments and emergency maintenance [61]. The purposeful sample has been identified as “trachoma at risk” communities based on annual trachoma screening that identifies active trachoma prevalence of 5% or greater among Aboriginal and Torres Strait Islander children aged 5-9 years in the last 5 years [62]. #endingtrachoma has been building the capacity of local Indigenous EHPs to enter homes, identify and audit trachoma risk factors (eg, ability to wash), fix minor plumbing issues, yarn with tenants about hygiene, and provide hygiene resources. The #endingtrachoma project revisits each house every 4 months. An important component of the project is to transfer the auditing skills to the EHPs who service these communities. These EHPs are contracted to visit these communities every 4 months and collect data using the mobile app. This research will build on this to include environmental health and food security risks.

### Indigenous Governance

A Research Governance Group to support and guide the research comprises Indigenous environmental health experts, a Department of Communities (Housing) representative, an Indigenous policy expert, and an Indigenous community advocate. These representatives live in the Midwest, Kimberley, Goldfields, and Perth or Peel regions of Western Australia. Reciprocity is reflected in this Research Governance Group, whose membership has been determined and agreed in consultation with different communities involved in the research. Roles of the group are to identify potential benefits and implications related to the values and aspirations of the community; avert any unintended consequences; provide advice on cultural security issues, barriers and enablers, data sovereignty and dissemination, and food security issues relevant to remote communities; and contribute to knowledge transfer strategies.

### Community Engagement

The established relationships developed with each community through the #endingtrachoma project inform and support this research methodology. All data collection is informed by community aspirations to ensure food security. Permission to enter each community will be obtained prior to, and with an invitation from, the community chairperson of each community. Posters describing the study will be displayed in the community store, at the health clinic and art center, to advise community members of the date and purpose of the visit. Visits will coincide with the #endingtrachoma project visits.

### Mixed Methods Approach

#### Overview

This mixed method study involves (1) an in-home digital healthy home kitchen functionality audit and food security check incorporating digital images where appropriate (n=197), (2) yarns (short interviews) with tenants (n=100) exploring their perspectives on food security issues and preferred kitchen design, and (3) store pricing survey using the digital Healthy Diets ASAP (Australian Standardized Affordability and Price) app [63].

#### Internal Environment—Community Housing and Kitchen Audits

##### Overview

Over 12 months, 197 households across 19 communities will be visited. No private or state-owned houses will be audited. Some tenants will be away, and not all houses will permit entry, and based on our previous experience, a 70% participation rate is achievable. The long-term engagement with the community and the support of EHP facilitate this. Nonparticipation is predominantly due to cultural reasons such as death in the family and lore. Where entry is allowed, the food security researchers will conduct the kitchen audit and a face-to-face yarn with an adult tenant (n=100). Researchers on this food security project have been working on the #endingtrachoma project for the past 5 years and have extensive knowledge of the remote communities involved and have built robust and genuine relationships with the community leaders, local service providers, and many of the community members.

In each community, an Indigenous EHP team member will lead the conversation to gain permission to enter each community-managed house. The EHPs are local people who live and work in remote communities. They hold a very strong understanding of the communities. With permission, they will introduce the food security researchers to the tenant, who will invite the householder to participate in the research. A project information sheet will be provided or read out if required, and signed consent obtained with the option to opt out.

##### Kitchen Audit

The food security researchers will conduct a digital healthy home kitchen functionality audit. This audit is based on the SafetyCulture Healthy Home app used in the #endingtrachoma project and is consistent with the HLP 4 criteria [44,64]. It will be a new tool developed for this research. Knowledge and experience from 5 years of completing the healthy home audits via the SafetyCulture app will guide the design of the kitchen app. The audit will assess cleanliness and functionality for food storage, preparation, and cooking in household kitchens. The audit captures issues associated with kitchen functionality, availability and state of cooking appliances, presence of cleaning and hygiene products, and kitchen requirements to prevent pests and vermin entering the house. The taps in the kitchen are turned on to ensure that both hot and cold water are available to the house. These details of the audit have been included as examples of auditing practices. The 64-item audit assesses evidence of pests, mold, waste disposal, basic utilities, kitchen, plumbing and electrical appliances (eg, oven, stove, refrigerator, freezer, ventilation, and other white goods), kitchen benches, cupboards and pantry, cooking utensils, cleaning and hygiene products, windows, and doors. A question is asked within the app to identify the number of people who slept in the house on the previous night. This provides an indication of crowding issues. Digital photographs are included in the audit data collection. The #endingtrachoma team will provide emergency plumbing fixes throughout the house, including the kitchen, as required. If the repair is unable to be carried out, the fault will be reported to the housing provider. A kitchen kit comprising household kitchen hygiene products and a small plunger will be offered to each house where a complete audit has occurred to thank tenants for their participation.

##### Yarning

Yarning is a conversational process that involves the sharing of stories and the development of knowledge and reflects Indigenous people’s emphasis on oral traditions [65,66]. Yarning can involve a range of fluid narrative strategies such as anecdotes, stories, and personal experiences [67]. Burke et al [68] suggest that this fluid style of questioning is more likely to reflect Indigenous peoples’ perspectives, as it is less confrontational. It is considered to be one of the most appropriate research methodologies to determine Australian Indigenous community members’ perceptions of their health needs and is considered culturally safe if conducted in a way that is respectful, cooperative, and supports cultural practice [68,69]. Yarning has been shown to foster connection, establish trust, and enable researchers to explore topics in greater detail [70]. Tenants will be invited to have a one-to-one yarn, or if preferred, a group yarn, about what their kitchen might look like if they had the opportunity to design it and how they like to prepare the food they eat. The yarns will occur inside the homes while the kitchen audit and healthy home audits are being conducted. In some cases, the Indigenous EHP will instigate the yarn, and in other cases, where the EHP is busy fixing a plumbing issue, the yarn will be led by the research team (non-Indigenous). Either way, an Indigenous person will always be in the house when the yarns occur. The yarning question will encourage flexibility and disclosure. The participant will be asked to choose where they would like to have the yarn, and a Voice Memos mobile telephone audio recording app will be used to record the yarn.

#### External Environment—Community Store Pricing Audits

As food insecurity is driven by a complex range of factors, such as inadequate income to support meal planning and food purchases, food prices, poor access to safe storage and cooking facilities, limited transport options to access stores, and poor access to fresh and nutritious foods, focusing on the community store environment and pricing policies is a critical component of this study. A recommendation from the 2009 Parliamentary Inquiry into remote Aboriginal and Torres Strait Islander community stores [28] was a requirement to display pricing. However, under Australian Consumer Law, there is no specific legal requirement for stores with less than 1000 m^2^ of floor space to display prices [71].

The Healthy Diets ASAP app [63] will be used to audit prices of food products (n=76) in each of the 15 independent stores located within the 19 remote Indigenous communities, along with audits in the 6 nearest regional towns and the capital city, Perth, for comparison. The digital survey tool has been tailored to assess the food affordability component of food security in remote Indigenous communities [72]. This Healthy Diets ASAP method allows the “assessment of price, price differential and affordability of healthy (recommended) and current (unhealthy) diets of Aboriginal and Torres Strait Islander people living in different locations with other population groups in Australia” [72]. Observation of evidence of pricing policies will also be undertaken, including the presence or absence of prices on shelves for all products. Photographs of food and hygiene items will be taken where appropriate and will confirm the presence of shelf pricing. Hygiene products (n=28) that are required to keep kitchens and work areas clean and support handwashing (eg, soap and detergent) will also be priced. Data will be collected in-store using an iPad and uploaded via a web-based portal. Permission will be sought from each community store manager to undertake the pricing audit, and entry to communities must be granted by the community chief executive officer (CEO) prior to each visit.

#### Reflexivity Statements and Addressing Potential Power Dynamics

Each researcher will include their reflexivity statement and procedures to support independent data verification to ensure the privileging of Indigenous peoples in the research process, which are outlined below. A number of activities are incorporated into the research to limit the potential for power dynamics and cultural differences to influence participant responses or researcher interpretations. Cultural mentors, elder and community members, and local Indigenous environmental health workers from each community assist with the process of continuous self-reflection. This reflection occurs during many stages of the project and ensures appropriate interpretation and communication between practitioners and community members. Indigenous researchers and advisors guide the research process and inform the timing and suitability of each visit to ensure that respect, reciprocity, and preservation of self-determination are central. Examples include:

- Data collection: Local Indigenous workers make approaches to houses and explain the research purpose. Non-Indigenous researchers are only invited to conduct interviews following tenants’ consent and work alongside Indigenous workers and community members. Research team members have long-term relationships with community workers who help create safe interview conditions [73]. The yarns occur while the team members are working in the house. These connections and relationships provide a culturally safe and sensitive data collection, and yarning is used to facilitate 2-way knowledge sharing [74].
- Data interpretation: Two-way knowledge sharing is facilitated through yarning and feedback on the interpretation of data during analysis with the Research Governance Group and stakeholders in each community. Feedback is sought on culturally appropriate reporting, with language and interpretation a key part. Reports are modified based on feedback, and this process is iterative.
- Dissemination: To facilitate advocacy, the data are shared directly with stores and community leaders and relevant decision-makers (eg, the CEO, the Environmental Health Contracted service, and the store manager), who can then use it directly for advocacy purposes and to guide how the research project can use it to support their advocacy priorities.

This study will produce benchmarks based on measures and perceptions of environmental dimensions [75] influencing food security, in this case, food prices, kitchens, and tenants’ perspectives.

### Analysis

#### Kitchen Audit

Descriptive statistics of the 64 items in the kitchen audit using the SafetyCulture Healthy Homes app will be reported. Where appropriate, photographic images will be analyzed to assess the number of food security attributes recorded related to the 6 dimensions of food insecurity [21].

#### Store Pricing

Descriptive statistics of the price and availability of the 97-item hygiene data will be reported and relatively adjusted to the nearest regional town and capital city. The data from the remote store food and sanitation products will be entered digitally into an iPad using a web-based portal. Store pricing data will be directly uploaded to the Healthy Diets ASAP web-based portal, and analysis will be undertaken in accordance with the Healthy Diets ASAP protocol that compares the price, availability, and affordability of unhealthy and healthy food based on average income and family composition in the community (see protocol for more details [63]). The mean estimated family household income against the cost of unhealthy and healthy diets will be calculated and compared between the nearest urban and regional stores.

#### Yarns

Digital transcriptions from each interview will be deidentified, and a thematic analysis will be undertaken, guided by the theory of Braun and Clarke [76]. Two researchers (MS and CMP) will review the data to create initial themes and codes, and a coding frame will be created to ensure consistency and support idea generation. Themes will be reviewed for the need to combine or consolidate through consensus. The themes and codes will be shared with the other researchers and the governance committee to ensure that cultural protocols are followed.

#### Two-Way Sharing of Information

The results will be shared with each community in a timely and culturally appropriate manner, with processes guided by the research governance committee. To support transparency, accountability, and advocacy, results will be provided in plain language, and graphical pricing survey reports will be provided directly to store managers, community leaders (to disseminate among community members and household owners), and the governance group via easy-to-read fact sheets. Information will include the comparison of the average price and percentage of income needed to purchase a healthy and unhealthy diet at the community store, regional town store, and capital city; a summary of the kitchen audits; and key messages from the yarns. Security and access controls around the Indigenous data collected will be applied in accordance with Indigenous cultural protocols [77,78]. Only data that have the potential to directly benefit the community will be collected, and the process recognizes that Indigenous people are the decision-makers for the access, control, and use of the data.

## Results

The project was funded in November 2024, and data collection commenced in February 2025. Accessing remote communities is challenging due to distance, a lack of transport options, weather restrictions, and costs. At the time of publishing, 10 remote communities had been accessed, with 47 yarns being recorded, 61 healthy kitchen audits being completed, and 8 remote store prices being analyzed. The final data collection is due to be completed by June 2026, and results will be analyzed by November 2026.

There are several expected outcomes associated with this research. The internal environmental health data collection will provide baseline information on the functionality of kitchens in the very remote communities that participate in this research. Broadly, the expected outcomes reported are shown in Textbox 1.

**Expected outcomes of the benchmarking environmental health influences on food security in very remote Indigenous communities in Western Australia.**

**Overall project**

- Number of remote communities providing permission for visits
- Number of kitchen audits attempted and completed
- Number of community-based advocacy campaigns associated with food security
- Advocacy to improve housing maintenance policy and timing for remote communities

**Internal audits**

- Number of completed kitchen audits by community
- Number of maintenance fixes to improve food security
- Number of nonfunctioning kitchen facilities
- Number of food security risks identified (per 64 items)
- Number of food security risks identified by food security dimensions
- Number of kitchen health hardware referrals or house by community
- Number of kitchen hygiene kits distributed or community
- Number of referrals to other agencies that focus on hygiene in the home project

**Yarns**

- Number of interviews
- Themes identified
- Number of ideas for future kitchen design

**External audits**

- The mean (SD) cost of the sanitation items
- The mean (SD) cost of the current diet
- The affordability of a healthy diet and the current diets
- Cost comparison of diet and sanitation items with the nearest town and the capital city

**Dissemination**

- Number of brief research or fact sheets by community
- Number of advocacy plans by community

The data generated as a result of this research will be prepared for dissemination and used to prepare targeted and community-led advocacy approaches to increase affordability, quality, and availability of food in remote communities; appropriate retail practices in remote community stores; improved remote house maintenance routines and priorities; the functionality of remote kitchens through plumbing fixes; and community-generated evidence on culturally appropriate kitchen design for remote community houses.

## Discussion

### Principal Findings

A key objective of this unique mixed methods, practice-informed study is to establish a benchmark for environmental health factors influencing household food security in remote Indigenous communities in Western Australia. Measuring and reporting on the 3 aspects of household food security together—household kitchen functionality, community kitchen preferences, and external retail practices—have not been previously undertaken.

Some previous research has addressed elements of this project. For example, Yashadhana et al [79] used participatory action research to explore eye health inequity in 4 remote Indigenous communities in the Northern Territory and north-western New South Wales. Their results identified that food insecurity was associated with economic disadvantage and its associated determinants including limited access to fresh, affordable food or a refrigerator to store food and medicine [79]. The findings also highlighted the importance of cultural sovereignty [79].

This study’s approach of quantifying environmental health food security drivers in 19 remote communities, guided by the Research Governance Group, will provide unique data to understand food access issues, kitchen functionality, community preferences for food preparation facilities, and preferred kitchen design. The house maintenance referral processes already in place through the #endingtrachoma program will ensure that issues related to the functionality of remote kitchens are either solved on site (eg, plumbing fixes and broken taps) or elevated to a higher priority to the appropriate service provider (eg, broken stoves and ovens and unsafe electrical points). These issues critically affect food security. The approach and the baseline data generated will be used by communities to advocate for improved housing design and maintenance schedules to ensure the timeliness of maintenance issues within the home that affect health.

Community preferences for kitchens and food access will be used to advocate for culturally appropriate co-designed kitchens and houses. The findings support the philosophy that a house is a social determinant of health rather than simply an asset. The findings will be shared with relevant government agencies and decision-makers including, for example, those responsible for remote housing, retail, community services, and remote health. The format for presenting the findings will be tailored to suit the audience and may include reports to government agencies, face-to-face advocacy meetings, conference presentations, meeting agenda items for discussion, and fact sheets. Support will be offered to community members to become involved in dissemination strategies as interest grows.

Environmental health–enabling factors in community stores influence individuals’ food choices. Bryce et al [80] have shown that the retail food environment influences the availability, cost, affordability, quality, safety, advertising, and promotion of food and can also influence consumer food choice and food security. This study builds on previous research to benchmark community store retail practices and food pricing surveys in remote Indigenous communities [6,7,72,81,82] and environmental health referral schemes to assist households to improve the environmental health determinants of trachoma [83]. This research works alongside the #endingtrachoma program, which was co-designed by Indigenous environmental health professionals, community health, local and state government staff, and academics. The research design incorporates the dissemination and proposed use of data by community members to advocate for more effective interventions and extends the #endingtrachoma service delivery model, which has been operated for the past 5 years [14].

Changes in the environmental health determinants of food insecurity are expected to reduce food insecurity, improve diet quality, and increase the likelihood of diet-related health benefits. This benchmarking study is likely to highlight suboptimal retail practices, such as the absence of prices on food items, and environmental health determinants of food insecurity, resulting in the need for additional advocacy efforts. This is the first time that sanitation items have been audited in remote locations, providing evidence to advocate for affordable products to protect families against diseases associated with poor hygiene.

This study uses digital tools to build on previous research linking health conditions to housing [47] and will add a contemporary benchmark of kitchen health hardware in very remote Australian communities [13,38,45]. Previous research stressed the importance of Indigenous families having adequately designed kitchens, both internal and external, to enable improved food security and maintain kinship [34]. Through both the kitchen audits and the yarns, this study will build knowledge about the environmental health determinants of food security in remote communities and support the development of strategies to address the issues identified [4,84]. A final strength of the methodology is that it prioritizes the voice of Indigenous people residing in very remote communities in Australia.

### Strengths and Limitations

Several factors may influence this research, including geographic isolation and distance to travel to each community, climatic conditions and weather events (eg, floods and fires), and cultural practices (eg, Lore and Sorry Time), which dictate periods when communities are not accessible. Knowledge formed through involvement with the #endingtrachoma project will inform the most appropriate seasons and times to visit communities. Communities will not be entered without explicit approval from the community CEO. Different geographic locations and cultural practices mean that the results of the kitchen audit and yarns are not generalizable to other communities or populations.

There are limitations related to the choice of food price measure, as the purpose of the Healthy Diets ASAP is to compare the price of a healthy diet versus an unhealthy dietary pattern and does not measure a wider selection of food products available, for example, where dietary preferences differ between communities. Time limitations in the current methodology would not permit the auditing of more food items or the quality of food, which is an issue impacting food security in remote communities.

The strength of this study is the research that is to be conducted in 4 remote regions in Western Australia and 19 of the 46 very remote stores in Western Australia. The benchmarking and methodology established may be used to further research in other communities. A further strength is that the store price benchmarking results will be compared within regions and to the nearest town and urban center to highlight food insecurity and high prices as potentially significant issues in remote communities. Being able to work alongside a well-known and experienced existing project that has strong connections into the communities to be visited and working with local Indigenous people in their communities are also strengths of this study.

### Conclusions

Household food security in remote communities in Australia is influenced by food affordability and the functionality of health hardware in remote housing, which impacts safe food preparation and storage. These conditions increase the risk of environmental health-related diseases among residents. Safe and suitable household kitchen infrastructure is essential to food security. Using digital technology and mixed methods, this research will provide local Indigenous communities, environmental health services, and stores with critical information to challenge decades of inaction on food insecurity in remote Australian communities. Benchmarking kitchen functionality and store pricing will provide transparent information to support targeted advocacy to improve access to safe, affordable, and nutritious food and the appropriate kitchen facilities to store and prepare it.

## Data Availability

The datasets generated or analyzed during this study are not publicly available due to data sovereignty requirements.

## Abbreviations

CEO: chief executive officer
CONSIDER: Consolidated Criteria for Strengthening the Reporting of Health Research Involving Indigenous Peoples
EHP: environmental health practitioner
Healthy Diets ASAP: Australian Standardized Affordability and Price
HLP: Healthy Living Principle

## References

[1] (2007). United Nations Declaration on the Rights of Indigenous Peoples.

[2] Commonwealth of Australia (2022). First Nations Vocabulary' using culturally appropriate language and terminology. Australian Public Service Commission.

[3] Mitchell T (2019). Colonial trauma: complex, continuous, collective, cumulative and compounding effects on the health of Indigenous peoples in Canada and beyond. Int J Indig Health.

[4] Ferguson M, Tonkin E, Brimblecombe J, Lee A, Fredericks B, Cullerton K, Mah CL, Brown C, McMahon E, Chatfield MD, Miles E, Cadet-James Y (2023). Communities setting the direction for their right to nutritious, affordable food: co-design of the remote food security project in Australian Indigenous communities. Int J Environ Res Public Health.

[5] Landrigan TJ, Kerr DA, Dhaliwal SS, Pollard CM (2018). Protocol for the development of a food stress index to identify households most at risk of food insecurity in Western Australia. Int J Environ Res Public Health.

[6] Pollard C, Landrigan T, Ellies P, Kerr D, Lester M, Goodchild S (2014). Geographic factors as determinants of food security: a Western Australian food pricing and quality study. Asia Pac J Clin Nutr.

[7] van Burgel E, Fairweather M, Hill A, Christian M, Ferguson M, Lee A, Funston S, Fredericks B, McMahon E, Pollard C, Brimblecombe J (2024). Development of a survey tool to assess the environmental determinants of health-enabling food retail practice in Aboriginal and Torres Strait Islander communities of remote Australia. BMC Public Health.

[8] (2022). Inquiry into food security in Australia: House of Representatives Standing Committee on Agriculture. National Aboriginal Community Controlled Health Organisation (NACCHO).

[9] (2025). Remote Aboriginal communities. Government of Western Australia, Department of Housing and Works.

[10] (2022). Western Australia: Aboriginal and Torres Strait Islander population summary. Australian Bureau of Statistics.

[11] (2024). Health and wellbeing of First Nations people. Australian Government: Australian Institute of Health and Welfare.

[12] (2024). National Aboriginal and Torres Strait Islander Health Survey. Australian Bureau of Statistics.

[13] (2023). Profile of First Nations people. Australian Institute of Health and Welfare.

[14] Pollard C, Farmar-Bowers Q, Millar J, Higgins V (2013). Selecting interventions for food security in remote Indigenous communities. Food Security in Australia.

[15] (2024). Goals 2: end hunger, achieve food security and improved nutrition and promote sustainable agriculture. The United Nations: Department of Economic and Social Affairs.

[16] (2001). The state of food insecurity and nutrition in the world. Food and Agriculture Organization of the United Nations.

[17] (2012). Coming to terms with terminology. Food and Agriculture Organization of the United Nations.

[18] Bowden M (2020). Understanding food insecurity in Australia. CFCA Paper No. 55. Australian Institute of Family Studies.

[19] (2025). National strategy for food security in remote Aboriginal and Torres Strait Islander communities. National Indigenous Australians Agency.

[20] (2019). IPC Technical Manual Version 3.0. Food and Agriculture Organization of the United Nations.

[21] Clapp J, Moseley WG, Burlingame B, Termine P (2022). Viewpoint: the case for a six-dimensional food security framework. Food Policy.

[22] (2012). Food security and nutrition: building a global narrative towards 2030. Committee on World Food Security: High Level Panel of Experts.

[23] (2020). Report on food pricing and food security in remote Indigenous communities: House of Representatives Standing Committee on Indigenous Affairs. The Parliament of the Commonwealth of Australia.

[24] Browne J, Walker T, Hill K, Mitchell F, Beswick H, Thow S, Ryan J, Sherriff S, Rossignoli A, Ropitini A, Johnstone M, Paradies Y, Backholer K, Allender S, Brown AD (2024). Food policies for Aboriginal and Torres Strait Islander health (FoodPATH): a systems thinking approach. Food Policy.

[25] Sherriff S, Kalucy D, Tong A, Naqvi N, Nixon J, Eades S, Ingram T, Slater K, Dickson M, Lee A, Muthayya S (2022). Murradambirra dhangaang (make food secure): Aboriginal community and stakeholder perspectives on food insecurity in urban and regional Australia. BMC Public Health.

[26] Godrich S, Davies C, Darby J, Devine A (2017). What are the determinants of food security among regional and remote Western Australian children?. Aust N Z J Public Health.

[27] Christidis R, Lock M, Walker T, Egan M, Browne J (2021). Concerns and priorities of Aboriginal and Torres Strait Islander peoples regarding food and nutrition: a systematic review of qualitative evidence. Int J Equity Health.

[28] (2009). Everybody's business: remote Aboriginal and Torres Strait community stores: House of Representatives Aboriginal and Torres Strait Islander Affairs Committee. The Parliament of the Commonwealth of Australia.

[29] Burns S, Bowser N, Smith J, Jancey J, Crawford G (2014). An exploratory study of smokers' and stakeholders' expectations of the implementation of a smoke-free policy in a university setting. Health Promot J Austr.

[30] Pratt S, DeMamiel M, Reye K, Huey A, Pope A (2014). Food security in remote Indigenous communities. The National Indigenous Australians Agency (NIAA).

[31] (2020). Inquiry into food pricing and food security in remote Indigenous communities. Parliament of Australia: House of Representatives Standing Committee on Indigenous Affairs.

[32] (2023). Hungry for change. Addressing food insecurity for children and young people affected by poverty. Parliament of Western Australia: Joint Standing Committee on the Commissioner for Children and Young People.

[33] (2023). Hungry for change: addressing food insecurity for children and young people affected by poverty: government response. Parliament of Western Australia: Standing Committee on the Commission for Children and Young People.

[34] Rajabipour A, Kutay C, Guenther J, Bazli M (2023). Factors to be considered in the design of Indigenous communities' houses, with a focus on Australian first nation housing in the Northern Territory. Dev Eng.

[35] Foster T, Hall NL (2021). Housing conditions and health in Indigenous Australian communities: current status and recent trends. Int J Environ Health Res.

[36] McKay FH, Haines BC, Dunn M (2019). Measuring and understanding food insecurity in Australia: a systematic review. Int J Environ Res Public Health.

[37] Bailie RS, Stevens M, McDonald EL (2012). The impact of housing improvement and socio-environmental factors on common childhood illnesses: a cohort study in Indigenous Australian communities. J Epidemiol Community Health.

[38] Towart R, Griew R, Murphy S, Pascoe F (2017). Remote housing review. Australian Government: National Indigenous Australians Agency.

[39] Altman J, Biddle N, Hunter B (2008). The challenge of 'Closing the Gaps' in Indigenous socioeconomic outcomes. Centre for Aboriginal Economic Policy Research.

[40] (2021). Reforms to meet Australia's future infrastructure needs: 2021 Australian infrastructure plan executive summary. Infrastructure Australia.

[41] Seemann K, Parnell M, McFallan S, Tucker S (2008). Housing for livelihoods: the lifecycle of housing and infrastructure through a whole-of-system approach in remote Aboriginal settlements. Desert Knowledge CRC.

[42] Campo M, Tayton S (2015). Domestic and family violence in regional, rural and remote communities. Australian Institute of Family Studies.

[43] Bailie RS, Wayte KJ (2006). Housing and health in Indigenous communities: key issues for housing and health improvement in remote Aboriginal and Torres Strait Islander communities. Aust J Rural Health.

[44] Pholeros P, Lea T, Rainow S, Sowerbutts T, Torzillo PJ (2013). Improving the state of health hardware in Australian Indigenous housing: building more houses is not the only answer. Int J Circumpolar Health.

[45] Torzillo PJ, Pholeros P, Rainow S, Barker G, Sowerbutts T, Short T, Irvine A (2008). The state of health hardware in Aboriginal communities in rural and remote Australia. Aust N Z J Public Health.

[46] (2024). 2.01 Housing. Australian Institute of Health and Welfare: National Indigenous Australians Agency: Aboriginal and Torres Strait Islander Health Performance Framework.

[47] Ali SH, Foster T, Hall NL (2018). The relationship between infectious diseases and housing maintenance in Indigenous Australian households. Int J Environ Res Public Health.

[48] Quilty S, Frank Jupurrurla N, Bailie RS, Gruen RL (2022). Climate, housing, energy and Indigenous health: a call to action. Med J Aust.

[49] Bailie R, Main N (2002). Environmental health survey year 2 evaluation. Indigenous Housing Authority of the Northern Territory.

[50] Kingsley J, Townsend M, Henderson-Wilson C, Bolam B (2013). Developing an exploratory framework linking Australian Aboriginal peoples' connection to country and concepts of wellbeing. Int J Environ Res Public Health.

[51] O'Rourke T, Nash D (2019). Aboriginal yards in remote Australia: adapting landscapes for Indigenous housing. Landsc Urban Plan.

[52] Page A, Memmott P (2021). Design: Building on Country.

[53] Grant E, Greenop K, Refiti A, Glenn D (2018). The Handbook of Contemporary Indigenous Architecture.

[54] Bailie R, Stevens M, McDonald E, Brewster D, Guthridge S (2010). Exploring cross-sectional associations between common childhood illness, housing and social conditions in remote Australian Aboriginal communities. BMC Public Health.

[55] Andersen MJ, Skinner A, Williamson AB, Fernando P, Wright D (2018). Housing conditions associated with recurrent gastrointestinal infection in urban Aboriginal children in NSW, Australia: findings from SEARCH. Aust N Z J Public Health.

[56] Huria T, Palmer SC, Pitama S, Beckert L, Lacey C, Ewen S, Smith LT (2019). Consolidated criteria for strengthening reporting of health research involving Indigenous peoples: the CONSIDER statement. BMC Med Res Methodol.

[57] (2018). Ethical conduct in research with Aboriginal and Torres Strait Islander peoples and communities: guidelines for researchers and stakeholders. National Health and Medical Research Council.

[58] (2023). Remoteness structure: Australian Statistical Geography Standard (ASGS) Edition 3. Reference period: July 2021-June 2026. Australian Bureau of Statistics.

[59] (2021). Socio-Economic Indexes for Areas (SEIFA), Australia. Australian Bureau of Statistics.

[60] (2018). Census of population and housing: characteristics of Aboriginal and Torres Strait Islander Australians. Australian Bureau of Statistics.

[61] Stoneham M, MacKenzie S (2019). #endingtrachoma Northern Goldfields Environmental Health Trachoma Project WA.

[62] Cowling C (2014). Australian trachoma surveillance report, 2013. Australian Governament: Department of Health, Disability and Ageing.

[63] Lee AJ, Kane S, Lewis M, Good E, Pollard CM, Landrigan TJ, Dick M (2018). Healthy Diets ASAP—Australian Standardised Affordability and Pricing methods protocol. Nutr J.

[64] (2025). iAuditor—inspection software & mobile inspection app. SafetyCulture.

[65] Rogers J (2018). Photostory and relatedness methodology: the beginning of an Aboriginal-Kanaka Maoli research journey (part one). Aboriginal Australian Stud J (forthcoming).

[66] Walker M, Fredericks B, Mills K, Anderson D (2014). "Yarning" as a method for community-based health research with Indigenous women: the Indigenous women's wellness research program. Health Care Women Int.

[67] Geia LK, Hayes B, Usher K (2013). Yarning/Aboriginal storytelling: towards an understanding of an Indigenous perspective and its implications for research practice. Contemp Nurse.

[68] Burke AW, Welch S, Power T, Lucas C, Moles RJ (2022). Clinical yarning with Aboriginal and/or Torres Strait Islander peoples—a systematic scoping review of its use and impacts. Syst Rev.

[69] Smith RL, Devine S, Preston R (2020). Recommended methodologies to determine Australian Indigenous community members' perceptions of their health needs: a literature review. Aust J Prim Health.

[70] Ayton D, Tsindos T, Berkovic D (2023). Qualitative Research—A Practical Guide for Health and Social Care Researchers and Practitioners.

[71] (2025). Unit pricing code. Australian Competition & Consumer Commission.

[72] Lee A, Lewis M (2018). Testing the price of healthy and current diets in remote Aboriginal communities to improve food security: development of the Aboriginal and Torres Strait Islander Healthy Diets ASAP (Australian Standardised Affordability and Pricing) methods. Int J Environ Res Public Health.

[73] Canuto K, Towers K, Riessen J, Perry J, Bond S, Ah Chee D, Brown A (2019). "Anybody can make kids; it takes a real man to look after your kids": Aboriginal men's discourse on parenting. PLoS One.

[74] Bessarab D, Ng'andu B (2010). Yarning about yarning as a legitimate method in Indigenous research. Int J Crit Indig Stud.

[75] Ryan N, Vieira D, Gyamfi J, Ojo T, Shelley D, Ogedegbe O, Iwelunmor J, Peprah E (2022). Development of the ASSESS tool: a comprehenSive tool to support rEporting and critical appraiSal of qualitative, quantitative, and mixed methods implementation reSearch outcomes. Implement Sci Commun.

[76] Braun V, Clarke V (2008). Using thematic analysis in psychology. Qual Res Psychol.

[77] (2023). Measuring what matters: submission to dept of treasury. National Aboriginal Community Controlled Health Organisation.

[78] (2023). Strategic directions 2023-2025. National Aboriginal Community Controlled Health Organisation.

[79] Yashadhana A, Fields T, Burnett A, Zwi AB (2021). Re-examining the gap: a critical realist analysis of eye health inequity among Aboriginal and Torres Strait Islander Australians. Soc Sci Med.

[80] Bryce S, Scales I, Herron L, Wigginton B, Lewis M, Lee A, Ngaanyatjarra Pitjantjatjara Yankunytjatjara Npy Women's Council (2020). Maitjara wangkanyi: insights from an ethnographic study of food practices of households in remote Australian Aboriginal communities. Int J Environ Res Public Health.

[81] Brimblecombe J, Ferguson M, McMahon E, Fredericks B, Turner N, Pollard C, Maple-Brown L, Batstone J, McCarthy L, Miles E, De Silva K, Barnes A, Chatfield M, Hill A, Christian M, van Burgel E, Fairweather M, Murison A, Lukose D, Gaikwad S, Lewis M, Clancy R, Santos C, Uhlmann K, Funston S, Baddeley L, Tsekouras S, Ananthapavan J, Sacks G, Lee A (2024). Benchmarking for healthy food stores: protocol for a randomised controlled trial with remote Aboriginal and Torres Strait Islander communities in Australia to enhance adoption of health-enabling store policy and practice. BMC Public Health.

[82] Lee AJ, Patay D, Summons S, Lewis M, Herron L, Nona F, Canuto C, Ferguson M, Twist A (2022). Cost and affordability of healthy, equitable and more sustainable diets in the Torres Strait Islands. Aust N Z J Public Health.

[83] Taylor HR, Anjou MD (2013). Trachoma in Australia: an update. Clin Exp Ophthalmol.

[84] Jaenke R, van den Boogaard C, McMahon E, Brimblecombe J (2021). Development and pilot of a tool to measure the healthiness of the in-store food environment. Public Health Nutr.

